# Nuclear contamination sources in surface air of Finnish Lapland in 1965–2011 studied by means of ^137^Cs, ^90^Sr, and total beta activity

**DOI:** 10.1007/s11356-019-05451-0

**Published:** 2019-05-24

**Authors:** Susanna Salminen-Paatero, Laura Thölix, Rigel Kivi, Jussi Paatero

**Affiliations:** 1 Present Address: Department of Chemistry, Radiochemistry, University of Helsinki, P.O. Box 55, FI-00014 Helsinki, Finland; 2 Finnish Meteorological Institute, P.O. Box 503, FI-00101 Helsinki, Finland

**Keywords:** ^137^Cs, ^90^Sr, Total beta activity, Nuclear weapon testing, Chernobyl, Fukushima

## Abstract

Radionuclides ^137^Cs and ^90^Sr and total beta activity were determined from air filters collected in Rovaniemi (Finnish Lapland) in 1965–2011. Nuclear contamination sources present in the air filter samples as well as temporal changes in radionuclide concentrations were examined. Ozone observations and meteorological modeling were used in combination with radionuclide analyses to study the reasons behind the observed seasonal concentration variation. In general, the magnitude and variation in activity concentrations of ^137^Cs and ^90^Sr and total beta activity in the surface air of Rovaniemi in 1965–2011 corresponded well with values from other countries. However, the obtained results prove in practice that hardly any refractory or intermediate radionuclides from the destroyed Chernobyl reactor fuel were introduced to Finnish Lapland. The main source of ^137^Cs and ^90^Sr and total beta activity in the surface air of Rovaniemi in 1965–2011 has been intense atmospheric nuclear weapon testing in 1950s–1960s and later tests performed in 1965–1980, as well as leakages from underground nuclear tests in Semipalatinsk, 1966, and Novaya Zemlya, 1987. For ^137^Cs and total beta activity, the influence of Chernobyl and Fukushima accidents was detected.

## Introduction

Artificial radionuclides in Arctic environment have been investigated during past decades and a great deal of knowledge about their concentrations and enrichment has been gained since the Arctic radioecology studies began in the 1960s (Miettinen et al. 1963; Hanson 1967; Kauranen and Miettinen 1969; Holm and Persson 1975, 1977; Jaakkola et al. 1978, 1981). However, concentrations in surface air and especially long-term time series of anthropogenic radionuclides have not been published to great extent. Beyond the journal articles, digitalized reports, and other available data, there is a lot of old, maybe forgotten data in reports from past decades that cannot be accessed or even found via common internet search engines.

In this study, air filter samples collected in Rovaniemi, Subarctic Finland in 1965–2011 were analyzed for determining the activity concentrations of ^137^Cs, ^90^Sr, total beta activity, ^238,239,240,241^Pu, and ^241^Am. The previous study of the same radionuclides in the surface air of Sodankylä (Finnish Lapland, about 100 km North-Northeast of Rovaniemi) was limited to 1 year, 1963, when the deposition maximum of nuclear weapon testing occurred (Salminen and Paatero 2009; Salminen-Paatero and Paatero 2012; Salminen-Paatero et al. 2012). The only anthropogenic nuclear contamination source was then the global fallout from nuclear weapon testing and a minor possibility of weapon-grade fallout.

Long-term time series (1965–2011) of ^137^Cs, ^90^Sr, total beta activity, ^238,239,240,241^Pu, and ^241^Am was produced in this study to observe the effects of different nuclear events in Finnish Subarctic atmosphere. Possible anthropogenic radionuclide contamination sources during the investigated time period include global fallout from nuclear weapon testing in the 1950s and the 1960s, SNAP-9A satellite accident (releasing ^238^Pu only), atmospheric nuclear weapon tests by China (the largest ones by China were made in 1967–1980), the Chernobyl nuclear accident in 1986, and the Fukushima nuclear accident in 2011. In addition, two cases of leaking underground nuclear tests are presented. The temporal changes in radioisotope concentrations and activity ratios were followed and used as indicators of radioactive contamination sources in Subarctic Finland. Furthermore, the underlying atmospheric factors causing a seasonal variation of radionuclide content of the air were studied with meteorological modeling and ozone observations.

## Experimental

### Air filter samples

The Finnish Meteorological Institute’s [FMI] Rovaniemi monitoring station (66°34′N, 25°50′E, elevation 198 m above sea level [a.s.l.]) is located at the Rovaniemi airport on the Arctic Circle. Weekly aerosol samples have been collected onto filters at Rovaniemi since April 1965. The filters are obtained from the instrumentation monitoring continuously aerosol beta activity (Paatero et al. 1994). The filter material is Whatman 42 paper. Weekly air volumes are about 1000 m^3^.

### Measurement of total beta activity from air filters

The total beta activity concentration of the air filters is measured about 5 days after the end of sampling. By that time, the short-lived ^222^Rn progeny has decayed to lead-210 and the ^220^Rn progeny has decayed to stable lead. The measured total beta activity thus consists of lead-210 and possible artificial beta emitters (Mattsson et al. 1996). The filters are stored in the FMI’s (Finnish Meteorological Institute’s) sample archive after the measurements.

### Measurement of ^137^Cs by gamma spectrometry from air filters

The activity of ^137^Cs in the air filters was determined by gamma spectrometry in 2013 and there were 2–48 years between the sampling period and gamma measurement. The air filters were combined to sets containing filters from 3 months to 1 year time period. The length of the combined sample time period was chosen based on the sampling year and estimated radioactivity concentration level in the surface air during that year. The filter sets were positioned to the edges of a Marinelli beaker of 1 l in the way that they surrounded evenly the beaker center. The activity of gamma-emitting radionuclides in the air filters was determined with GX 8021 HPGe gamma spectrometer (Canberra) using Genie 2000 Gamma Acquisition & Analysis program (Canberra). Typical counting time was 3 days per sample set. The counting efficiency of the gamma spectrometers was determined by measuring in-house standards, i.e., filter sets containing known amount of gamma emitters. These calibration samples were prepared by adding a known amount of Multinuclide standard solution No. 7503 (Eckert & Ziegler, Valencia, California) containing gamma emitters with energy range of 47–1333 keV, to clean paper filters homogeneously as fine droplets. The spiked filters were positioned evenly as stacks inside the Marinelli beaker similarly with the real air filter samples. As the calibrations for varying filter number were needed, from 3 months to 1 year time period, the spiked air filters were mixed with clean filters if needed for obtaining the correct number of air filters per calibration sample. After measuring the multi-gamma calibration filter sets, the efficiency and energy calibrations were executed with Genie 2000 Gamma Acquisition &Analysis program and the measured calibration spectra were fitted with dual polynomial option.

A subset of filters was measured individually or as pooled 4-week samples. These measurements were carried out with a 30% relative efficiency HPGe detector, a HV supply and a linear amplifier (Canberra Industries Inc.), a PCA3 computer add-on board multi-channel analyzer, and a GammaTrac™ spectrum analysis software (Oxford Instruments Inc.).

### Radiochemical separation procedures

**Fig. 1:**
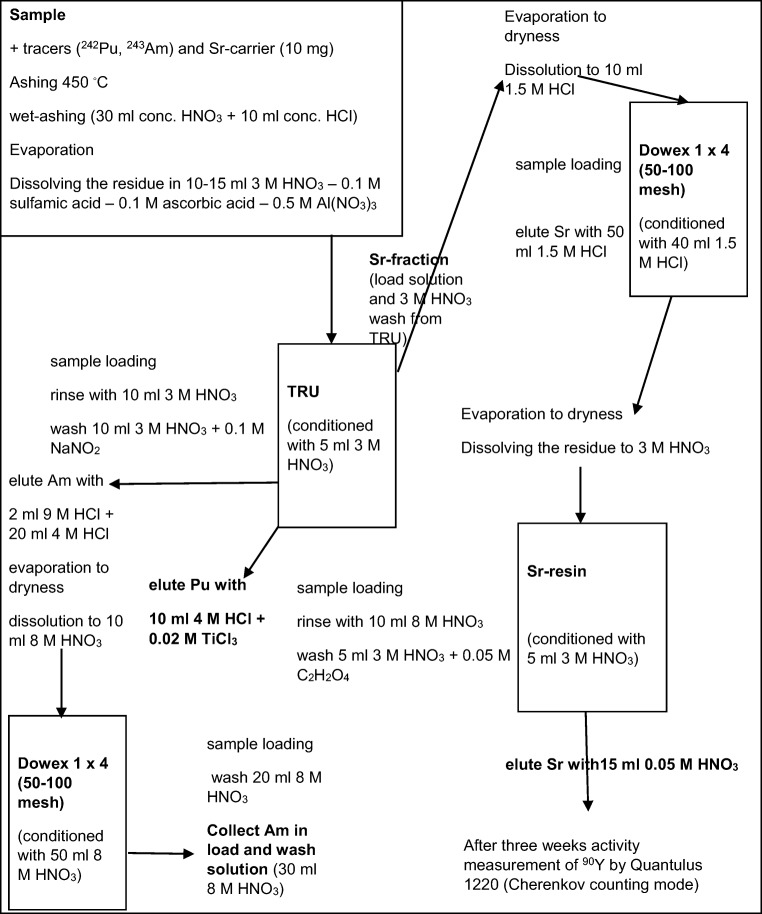
The separation method for ^90^Sr (and ^238,239,240^Pu and ^241^Am) from air filters

The detailed analytical scheme of the separation method is presented in Fig. 1. After gamma measurements, the air filters were analyzed as filter sets including sampling time period from 3 months to 5 years. For radiochemical separation of ^90^Sr, ^238,239,240^Pu, and ^241^Am, the filters were cut into pieces and ashed in an oven at 450 °C. The ash was digested with concentrated acids HCl and HNO_3_. Sr-carrier solution, ^242^Pu, and ^243^Am tracer solutions were added to the samples before digestion for yield determination. The samples were heated on a hot plate to almost boiling for 6 h and during the last hour, 1–2 ml of H_2_O_2_ was added to the samples for ensuring the complete oxidation of organic matter in the samples. The sample solutions were filtered and the solid residue was discarded. The filtered sample solutions were evaporated to dryness, 2 ml of conc. HNO_3_ was added and the samples were re-evaporated. From these residues, radionuclides ^90^Sr, ^238,239,240^Pu, and ^241^Am were separated with Dowex 1×4 anion exchange resin (Sigma Aldrich) and extraction chromatography resins TRU® and Sr resin® (TrisKem International). The basis of the separation method was extraction chromatography procedure described in Salminen and Paatero (2009) and it was completed with anion exchange steps.

The activity concentrations of ^238,239,240^Pu and ^241^Am in the air filter samples were determined by alpha spectrometry and the mass ratio of ^240^Pu/^239^Pu was measured by mass spectrometry. Discussion about the performance of radioanalytical method, as well as the activity concentrations and isotope ratios of ^238,239,240^Pu and ^241^Am in surface air of Rovaniemi will be published separately.

### Determination of ^90^Sr by LSC (liquid scintillation counting)

The activity concentration of ^90^Sr in the separated fractions (in 15 ml of 0.05 M HNO_3_) was determined with low-background liquid scintillation counter Quantulus 1220 (former Wallac, Perkin Elmer, Turku, Finland) via the activity concentration of daughter nuclide ^90^Y in the Cherenkov counting mode. The counting time was 10 h per sample. The counting efficiency correction for the ^90^Sr activity in the samples was performed by using a ^90^Y/^90^Sr standard sample series (Stamoulis et al. 2007) and this method produced a fixed counting efficiency value of 65% for Quantulus 1220. Chemical yield of Sr was determined with ICP-OES, being 41–98%.

## Results and discussion

### Activity concentration of ^137^Cs, ^90^Sr, and total beta activity in air filters

#### ^137^Cs

**Fig. 2:**
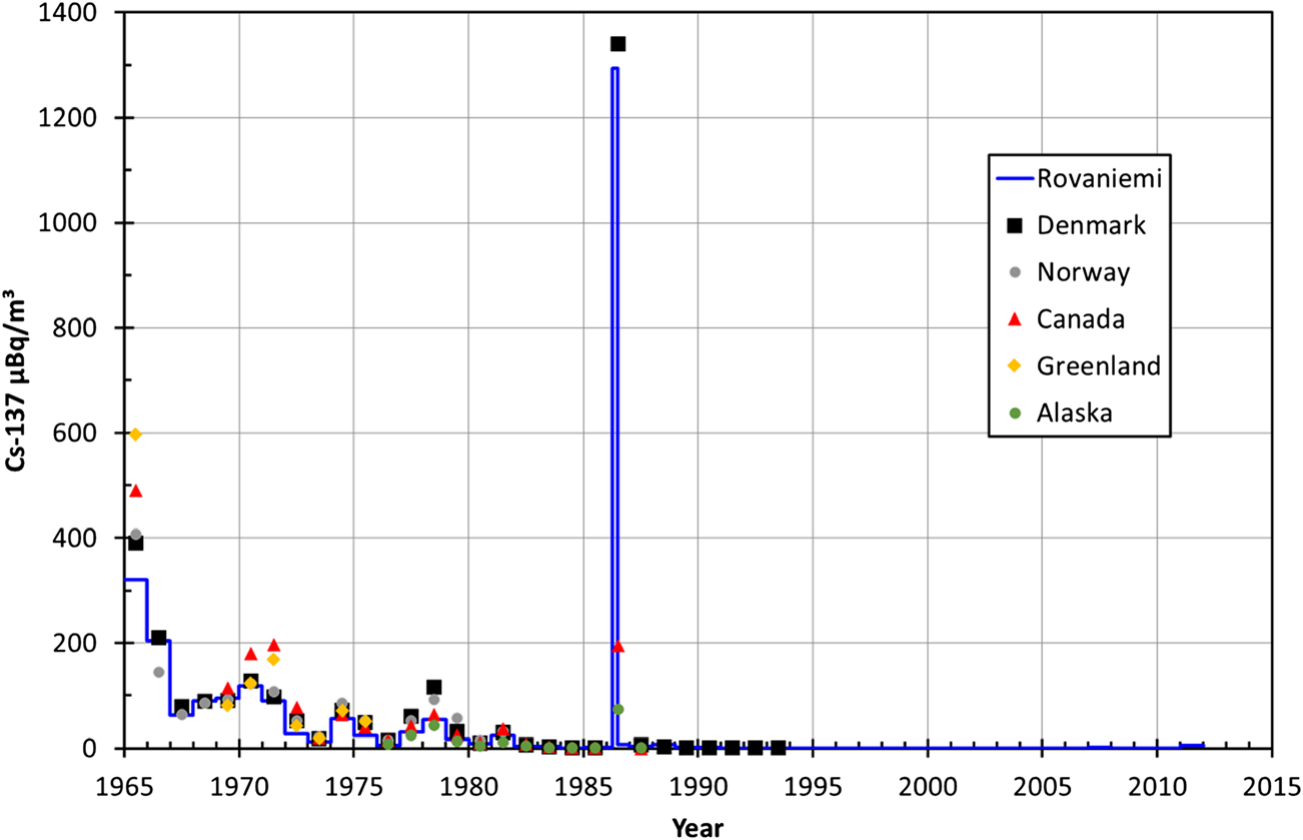
The activity concentration of ^137^Cs in surface air of Rovaniemi (μBq/m^3^) in 1965–2011 with corresponding values from Norway (Bergan 2002), Risö, Denmark (Aarkrog et al. 1995), Thule, Alaska, and Ontario (EML database 2000)

Between 1965 and 2011, the activity concentration of ^137^Cs had the highest value in 1965 (320±3 μBq/m^3^) and even higher in April–June 1986 (1294±7 μBq/m^3^) in surface air of Rovaniemi (Fig. 2, Table 1). The variation in concentration level of ^137^Cs resembles other studies in Northern hemisphere where the atmospheric activity concentration peaks occur in 1963 (the deposition maximum from atmospheric nuclear weapon testing) and in 1986 (the Chernobyl accident) (Aarkrog et al. 1995; Pommé et al. 1998; EML database 2000). For comparison, the activity concentration of ^137^Cs in 1963 was even as high as < 50–13,800±2700 mBq/m^3^ (average 2014±107 mBq/m^3^) in Sodankylä, Finnish Lapland, during 1963 (Salminen-Paatero and Paatero 2012), the activity concentration being orders of magnitude higher in 1963 than in 1965 in Finnish Lapland. The activity concentration of ^137^Cs was at the same level in Italy 1070 μBq/m^3^ (de Bortoli et al. 1966), Norway 407 μBq/m^3^ (Bergan 2002), Denmark 390 μBq/m^3^ (Aarkrog et al. 1995), Belgium ~ 500 μBq/m^3^ (Pommé et al. 1998), in 1965. These fore mentioned locations have been selected due to long-term and continuous observation data available.

**Table 1:** The activity concentrations of ^137^Cs, ^90^Sr, and total beta activity with the activity ratio of ^90^Sr/^137^Cs. The activity concentrations of ^137^Cs and ^90^Sr have been decay-corrected to the reference date, which is in the middle of the sampling period. Uncertainty of the values is one sigma error

Sampling time	A ^137^Cs (μBq/m^3^)	A ^90^Sr (μBq/m^3^)	A total beta (μBq/m^3^)	A ^90^Sr/A ^137^Cs
1965	320 ± 3	145 ± 30	2240	0.45 ± 0.09
1966	204 ± 2	86 ± 18	5070	0.42 ± 0.09
1967	62.9 ± 1.4	30 ± 6	630	0.47 ± 0.10
1968	89.4 ± 1.7	36 ± 7	1430	0.40 ± 0.08
1969	93.9 ± 1.7	37 ± 8	2250	0.40 ± 0.08
1970	119 ± 2	51 ± 10	2830	0.43 ± 0.09
1971	89.5 ± 1.1	41 ± 8	2660	0.46 ± 0.10
1972	28.0 ± 0.9	13 ± 3	790	0.45 ± 0.09
1973	11.4 ± 0.4	6.3 ± 1.3	220	0.55 ± 0.12
1974	56.6 ± 1.4	26 ± 5	1460	0.46 ± 0.09
1975	24.4 ± 0.8	12 ±3	620	0.50 ± 0.11
1976	5.7 ± 0.4	2.7 ± 0.6	290	0.48 ± 0.10
1977	30.5 ± 0.9	12 ± 3	1000	0.40 ± 0.08
1978	55.0 ± 1.1	28 ± 6	930	0.51 ± 0.11
1979	16.4 ± 0.3	2.8 ± 0.6	220	0.17 ± 0.04
1980	8.2 ± 0.3	3.7 ± 0.8	280	0.45 ± 0.09
1981	23.3 ± 0.5	10 ± 2	990	0.44 ± 0.09
1982	3.9 ± 0.3	0.54 ± 0.11	150	–
1983	3.2 ± 0.2		250	
1984	< 0.71		270	
January 1985–March 1986	1.7 ± 0.1		240	
April–June 1986	1294 ± 7	5.2 ± 1.1	7410	0.0040 ± 0.0008
July–December 1986	7.8 ± 0.3	< 0.19	140	–
1987	4.2 ± 0.3	< 0.03	260	–
1988	7.7 ± 0.4		260	
1989	2.8 ± 0.2		190	
1990	1.9 ± 0.1		220	
1991	< 0.52	< 0.02	240	–
1992	< 0.62		250	
1993	< 0.55		240	
1994	< 0.58		240	
1995	< 0.51		230	
1996	< 0.55	< 0.011	260	–
1997	< 0.47		250	
1998	< 0.47		280	
1999	< 0.45		280	
2000	< 0.49		240	
2001	< 0.45	< 0.010	280	–
2002	< 0.44		230	
2003	< 0.48		230	
2004	< 0.46		190	
2005	< 0.43		190	
2006	< 1.1	< 0.011	200	–
2007	< 1.2		210	
2008	< 0.94		170	
2009	< 1.1		220	
2010	< 1.0		280	
2011	5.4 ± 1.5	< 0.12	230	–

– indicates that the result is under detection limit

Attention has to be paid to sampling periods and lengths while comparing the activity concentration values after the Chernobyl accident in 1986. Different sampling periods, sampled air volumes, and calculation methods produce significantly different values that cannot be compared directly with each other. A good example about the effects of sampling dates and calculation methods on the concentration values can be learned from an Austrian study where the concentrations of radionuclides in surface air before and after 2 May 1986, was investigated at four sampling sites (Irlweck et al. 1993). It was observed that the fractions of ^137^Cs before and after 2 May 1986 had remarkable variation inside Austria: the percentage fractions of ^137^Cs activity concentration before 2 May and 2–5 May were 79% and 21% in Vienna, 68% and 32% in Linz, and 40% and 60% in Salzburg, respectively. The integrated activity concentration of ^137^Cs in Austria was 166–485 Bq/m^3^, depending on the sampling site, for the entire investigated period of 28 April–10 May 1986. The extremely high values of Austria are probably due to sampled small air volumes together with individual hot particles presumably present in these air samples. These two factors together produce excessively high radionuclide concentration values.

The pooled air filter sample of Rovaniemi in April–June 1986 has same activity level of ^137^Cs with Risö, Denmark where the arithmetic mean value for the year 1986 was 1340 μBq/m^3^ (Aarkrog et al. 1995). A much higher value, monthly average of about 100 mBq/m^3^, has been reported in Belgium in 1986 (Pommé et al. 1998). A year average value for ^137^Cs concentration in 1986 was 196 μBq/m^3^ in Ontario, Canada, being lower than European values, but on the other hand, monthly value for May 1986 in Ontario was as high as in Europe, 1900 μBq/m^3^ (EML database 2000). This case shows again the importance of calculation method for activity level magnitude and further considerations.

Based on these activity concentration values, the activity concentration of ^137^Cs in Rovaniemi has quite similar trends and level during these almost five decades as in other European countries and Arctic areas.

#### ^90^Sr

**Fig. 3:**
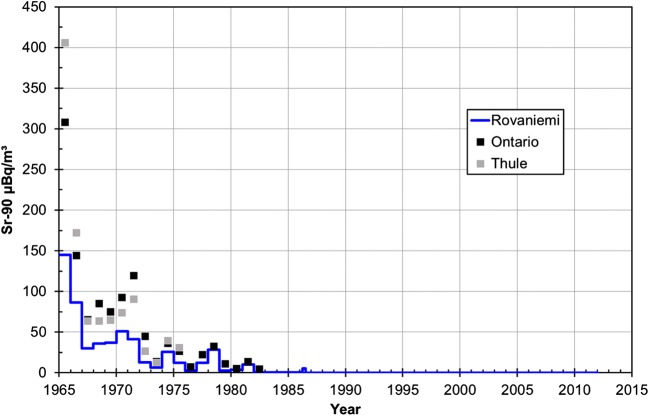
The activity concentration of ^90^Sr in surface air of Rovaniemi (μBq/m^3^) in 1965–2011 with corresponding values from Thule and Ontario (EML database 2000). The activity concentrations in Rovaniemi were below MDA since summer 1986. The value “half of MDA” has been used for those samples as activity concentration

The activity concentration of ^90^Sr was < 0.01 μBq/m^3^–145 ± 30 μBq/m^3^ in surface air of Rovaniemi during 1965–2011. The lowest value occurred in 1996–2010 and the highest in 1965 (Table 1, Fig. 3). In general, the atmospheric concentration of ^90^Sr has been constantly decreasing in Rovaniemi after 1965. In 1963 occurred both the atmospheric deposition maximum and start of the partial nuclear test ban treaty. The air sampling started 2 years later in Rovaniemi in 1965. By that time, the majority of ^90^Sr from atmospheric nuclear weapon testing that was executed before 1963 has been deposited to the ground. For comparison, the activity concentration of ^90^Sr was < 10–5340±290 mBq/m^3^ (average 1250±80 mBq/m^3^) in Sodankylä, Finnish Lapland in 1963 (Salminen-Paatero and Paatero 2012). The activity concentration of ^90^Sr in Lapland has been about 10,000-fold in the year of deposition maximum 1963 compared to year 1965.

The surface air activity concentrations of ^90^Sr in Rovaniemi are in agreement with other values from year 1965 to March 1986 showing similar decreasing trend during that time. The highest activity concentration 145±30 μBq/m^3^ in 1965 is at the same magnitude as other values from 1965 by de Bortoli et al. (1966), Aarkrog et al. (1995), and EML database (2000). Similar decreasing trend in activity concentration of ^90^Sr from 1965 to March 1986 has been observed in Risö (Aarkrog et al. 1995), in Ontario until end of sampling in 1985, and in Thule until end of sampling in 1976 (EML database 2000).

After April–June 1986 (5.2±1.1 μBq/m^3^), the activity concentration of ^90^Sr was under the limit of detection. Based on the varying activity concentration level through decades, ^90^Sr in the surface air of Finnish Lapland originates mostly from the old atmospheric nuclear weapon testing fallout. Only minor contribution from the Chernobyl NPP and later atmospheric nuclear weapons tests in 1965–1980 can be seen in the concentration variation of ^90^Sr (Fig. 3, Table 1). The Chernobyl-derived ^90^Sr was more evident in Risö, Denmark where the activity concentration of ^90^Sr in air was ~ 100 μBq/m^3^ in 1986 (Aarkrog et al. 1995). The initial plume from the exploded reactor of Chernobyl contained refractory elements and intermediate elements like ^90^Sr, and that plume did not reach Finnish Lapland. In the second plume, more volatile elements including ^137^Cs, were distributed and that plume passed Finnish Lapland.

#### Total beta activity in surface air of Rovaniemi in 1965–2011

**Fig. 4:**
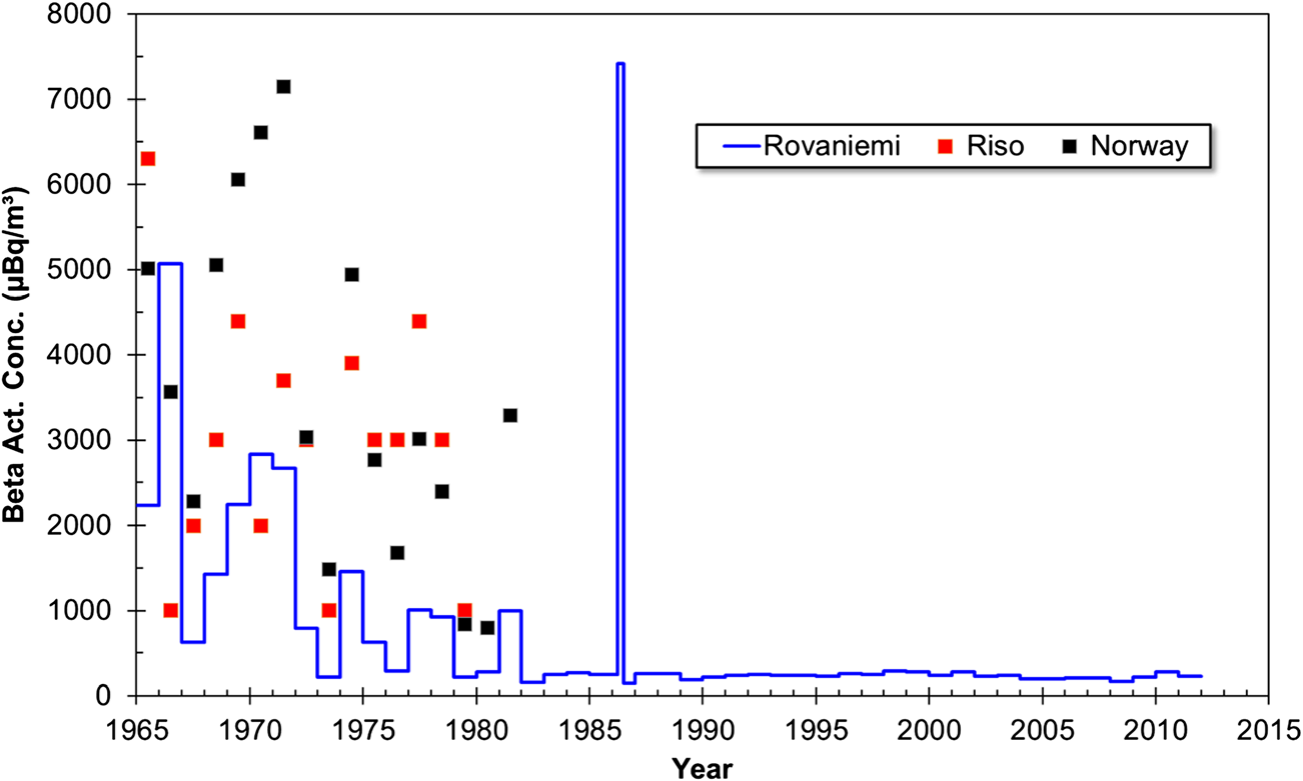
The total beta activity concentration in surface air of Rovaniemi (μBq/m^3^) in 1965–2011 with corresponding values from Norway (Bergan 2002) and Risö, Denmark (Aarkrog et al. 1995)

Total beta radioactivity in surface air of Rovaniemi had the lowest value 140 μBq/m^3^ in July–December 1986 and the highest value 7410 μBq/m^3^ in April–June 1986 (Table 1, Fig. 4). The highest value is due to the Chernobyl accident but other high values for total beta activity occur in 1965–1966, 2–3 years after the deposition maximum from atmospheric nuclear weapon testing, and later 1969–1971 due to leaked underground nuclear weapon test of 3 Mt. executed by China on 27.12.1968. Similar trends and total beta activity levels have been observed in Norway (Bergan 2002), Denmark (Aarkrog et al. 1995), and Belgium (Pommé et al. 1998). However, total beta activity concentration in 1986 after the Chernobyl accident was significantly lower in Rovaniemi compared to values of 1,000,000 μBq/m^3^ or even higher, measured in Southern Finland (Paatero et al. 2010b), Bulgaria (Paatero et al. 2010b), and Belgium (Pommé et al. 1998). The high total beta activity concentration in 1986 after the Chernobyl accident is mainly due to ^131^I and other short-lived nuclides present in the exploded reactor material. In our previous study about Finnish Lapland, total beta activity of 1850–477,300 μBq/m^3^ (average 80,770 μBq/m^3^) was measured in Sodankylä in 1963, the year of the atmospheric deposition maximum (Salminen-Paatero and Paatero 2012).

### Activity ratios ^90^Sr/^137^Cs and total beta/^137^Cs

**Fig. 5:**
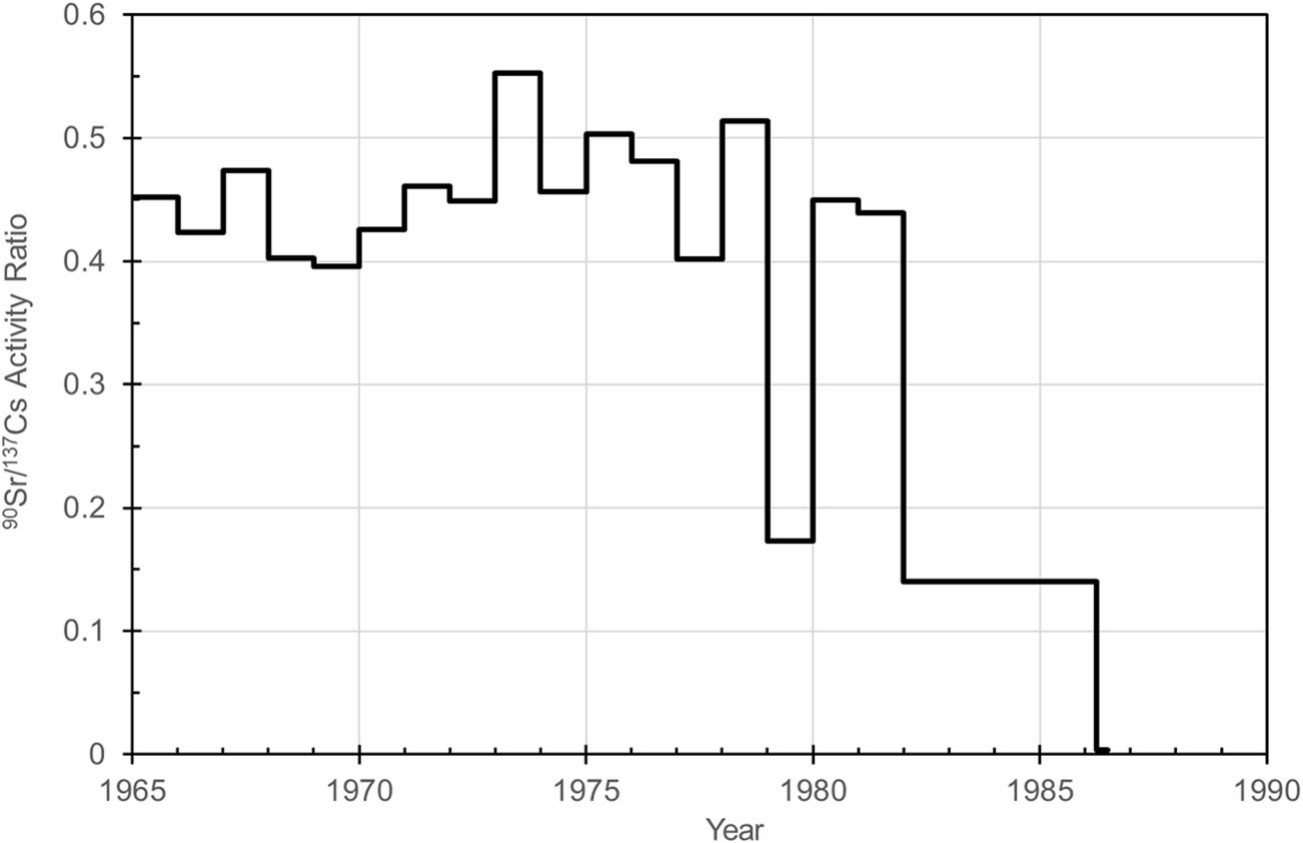
The activity ratio of ^90^Sr/^137^Cs in surface air of Rovaniemi during 1965–June 1986. After June 1986, the activity concentration of one or both isotopes was below the detection limit

The activity ratio ^90^Sr/^137^Cs was 0.40–0.55 in surface air of Rovaniemi in 1965–1981, with one deviating value 0.17 in 1979 (Table 1, Fig. 5). These ratio values of Rovaniemi are a bit lower than reported activity ratios 0.600–0.725 due to global fallout from nuclear weapon testing (UNSCEAR 1966; de Bortoli et al. 1966). After April–June 1986, either one or both isotopes had activity concentration below detection limit. The activity ratio ^90^Sr/^137^Cs had the lowest value in April–June 1986, 0.0040±0.0008. This ratio value is extremely low and it confirms the earlier discussed existence of Chernobyl-derived ^137^Cs and absence of Chernobyl-derived ^90^Sr in Rovaniemi, as well as previously suggested occurrence of ^90^Sr and plutonium in the same contamination plumes from the exploded Chernobyl nuclear reactor to Finland (Paatero et al. 2010a). About 85 PBq of ^137^Cs and 10 PBq of ^90^Sr were released during the Chernobyl accident (UNSCEAR 2000), producing ^90^Sr/^137^Cs activity ratio of 0.118 for Chernobyl-derived ^90^Sr and ^137^Cs contamination, assuming similar atmospheric migration of both isotopes. The post-Chernobyl ratio value of Rovaniemi is orders of magnitude lower than this Chernobyl-derived ratio value. Previously activity ratio ^90^Sr/^137^Cs has been determined to be 0.08±0.03–1.46±0.51 (average 0.51±0.01) in Sodankylä, Finnish Lapland, in 1963 (Salminen-Paatero and Paatero 2012).

Activity ratio total beta/^137^Cs was between 6 and 375 in Rovaniemi in 1965–1990. The lowest value occurred in April–June 1986 when there was substantial amount of Chernobyl-derived ^137^Cs in the air filter samples. The highest value for the ratio was in 1984 when the air filter samples contained aged nuclear weapon testing fallout. Similar variation in total beta/^137^Cs activity ratio has been observed in Sodankylä, Finnish Lapland, during 1 year, 1963. Then the ratio was 2.3–314 (average value 43) (Salminen-Paatero and Paatero 2012).

### Selected nuclear incidents: observations in Rovaniemi

#### Case “The leakage from Semipalatinsk, 1966”

The Soviet Union performed an underground nuclear test at Semipalatinsk test site 18 December 1966 04:58 UTC according to the seismographical observations. Part of the produced fission products, especially gaseous and volatile nuclides, leaked into the atmosphere causing a significant fractionation of the fission product mixture. A stable high-pressure area was located over Central Russia and the anti-cyclonal flow around the high-pressure center brought the polluted air masses to Finland. The radioactivity level in the surface air in Finland began to rise in the morning of 21 December 1966 (Kauranen et al. 1967).

**Fig. 6:**
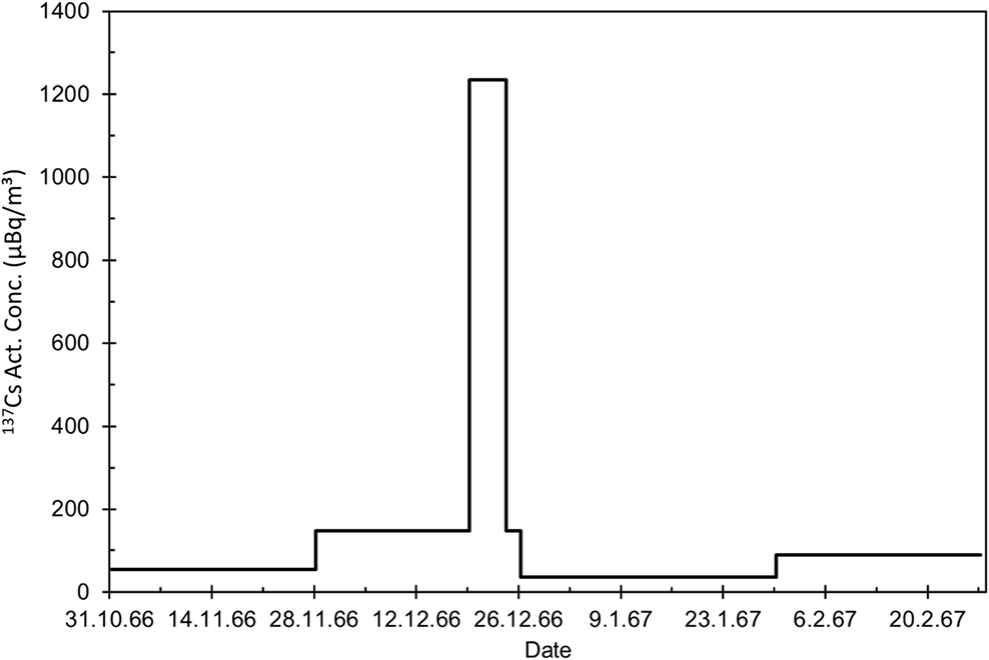
The activity concentration of ^137^Cs (μBq/m^3^) in weekly air filters from Rovaniemi in 31.10.1966–28.2.1967, measured before combining the filters for pooled samples

An order of magnitude increase in the ^137^Cs activity concentration was observed in Rovaniemi as the emission plume crossed Northern Finland (Fig. 6). The increase was even bigger in the case of total beta activity concentration, two orders of magnitude (Finnish Meteorological Office 1967). This was due to the relative abundance of volatile fission products and fission products with gaseous precursors compared with ^137^Cs. The observed weekly maximum concentration was the highest ever recorded in Rovaniemi and only ca. 30% less than the highest concentrations observed in Finland in 1961–1963, i.e., during the era of intense atmospheric nuclear testing.

#### Case “The Chernobyl accident, 1986”

The 1986 Chernobyl nuclear accident happened in the Soviet Union, current Ukraine (International Atomic Energy Agency 1986). The initial emission plume spread to Southern Finland within a couple of days. A frontal zone over Central Finland route first hindered the emission plume to reach Northern Finland. The emissions arrived at Northern Finland a few days later but the activity concentrations in the ground-level air were only a few per mille of those observed in Southern Finland (Paatero et al. 2010a).

**Fig. 7:**
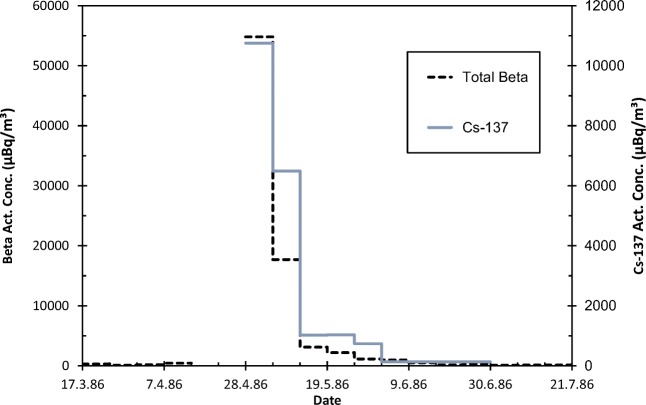
Weekly activity concentrations of total beta activity (μBq/m^3^, left y-axis) and ^137^Cs (μBq/m^3^, right y-axis) in Rovaniemi 17.3.–21.7.1986

The weekly total beta activity concentration of surface air in Rovaniemi rose from the natural level of a few hundred to 55,000 μBq/m^3^ during the first week of May 1986 (Fig. 7). This is only a quarter of the activity concentration observed in December 1966. The ^137^Cs activity concentration in May–June 1986 (Fig. 7) corresponds to an annual exposure to 400 μBq/m^3^. This value is close to the average ^137^Cs activity concentration in 1965. The contamination from the Chernobyl accident was quickly, mostly within a month, removed from the troposphere as there was no stratospheric reservoir to supply more contamination to the troposphere.

#### Case “The leakage from Novaya Zemlya, 1987”

An underground nuclear test was conducted at the Novaya Zemlya test site, Soviet Union, 2 August 1987. The yield of the detonation was 150 kt. A radioactive release into the surface air occurred 1.5 min after the explosion. All the staff was immediately evacuated from the area with helicopters, contrary to a similar accident in October 1969 leading to high radiation doses to test personnel (Khalturin et al. 2005). Thus, no cases of acute radiation sickness occurred. According to atmospheric dispersion model calculations, the contaminated air masses moved South-Westwards towards Kola peninsula and Northern parts of Finland, Norway, and Sweden. In Finland, the plume was first detected in an aerosol sample collected at Ivalo, 240 km NNE of Rovaniemi 6–7 August 1987, i.e., 4 days after the incident.

**Fig. 8:**
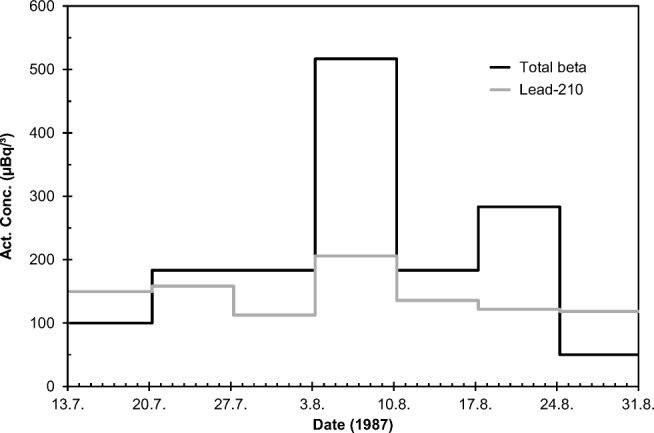
Total beta and ^210^Pb activity concentrations (μBq/m^3^) in Rovaniemi in July–August 1987

The recordings of aerosol beta activity and ^210^Pb activity concentrations reveal a clear presence of non-natural radioactivity in the air in Rovaniemi between 3 and 10 August 1987 (Fig. 8). The measurement of ^210^Pb is based on alpha counting of the ingrown daughter nuclide ^210^Po (Mattsson et al. 1996). The ^210^Pb results are from Sodankylä, but they are representative also for Rovaniemi because the ^210^Pb content of the air varies little in the scale of a few hundred kilometers. Observations of airborne iodine-131 in Rovaniemi resemble these recordings (Bjurman et al. 1990).

#### Case “The Fukushima accident, 2011”

A tsunami caused by a huge earthquake east of the Honshu Island, Japan on 11 March 2011 hit the Fukushima Dai-ichi nuclear power plant causing releases of radioactivity to both the atmosphere and the ocean. Within 2–3 weeks, the emissions spread practically all over the Northern hemisphere (Paatero et al. 2012).

**Fig. 9:**
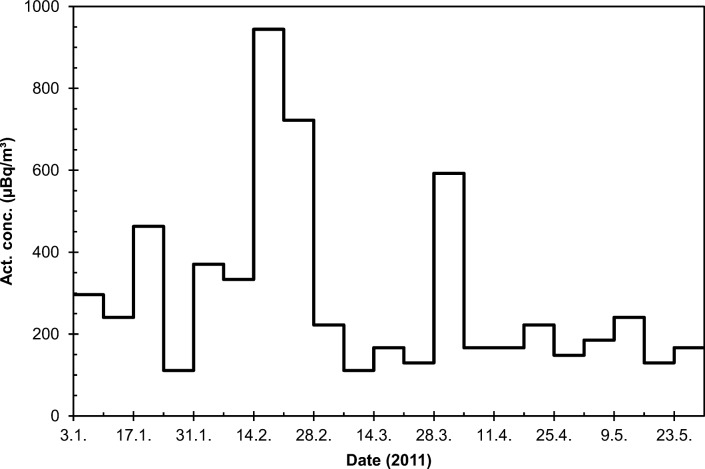
Total beta activity concentration (μBq/m^3^) in Rovaniemi in January–May 2011

**Fig. 10:**
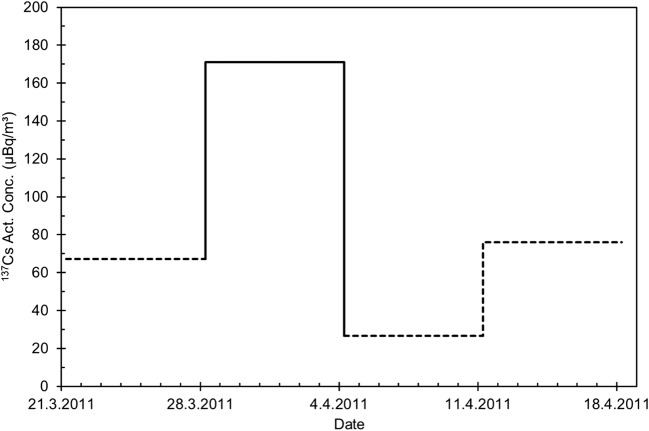
The activity concentration of ^137^Cs (μBq/m^3^) in weekly air filters from Rovaniemi in 21.3.–18.4. 2011, measured before combining the filters for pooled samples. The dotted parts of the curve represent “half of MDA” concentrations

The total beta activity concentration at Rovaniemi did not rise to unusual levels (Fig. 9). Higher values are frequently observed due to natural radioactivity (^210^Pb), as is evident from the observations in February 2011, 1 month before the Fukushima accident. The ^137^Cs activity concentration remained below detection limit excluding the week 28 March–4 April. During this week, a ^137^Cs activity concentration of 170 μBq/m^3^ (± 18%) was observed (Fig. 10). This is in agreement with the average activity concentration of 80 μBq/m^3^ observed in Rovaniemi city (Mustonen 2012).

#### “Atmospheric nuclear tests 1965–1980 and airborne ^137^Cs”

**Fig. 11:**
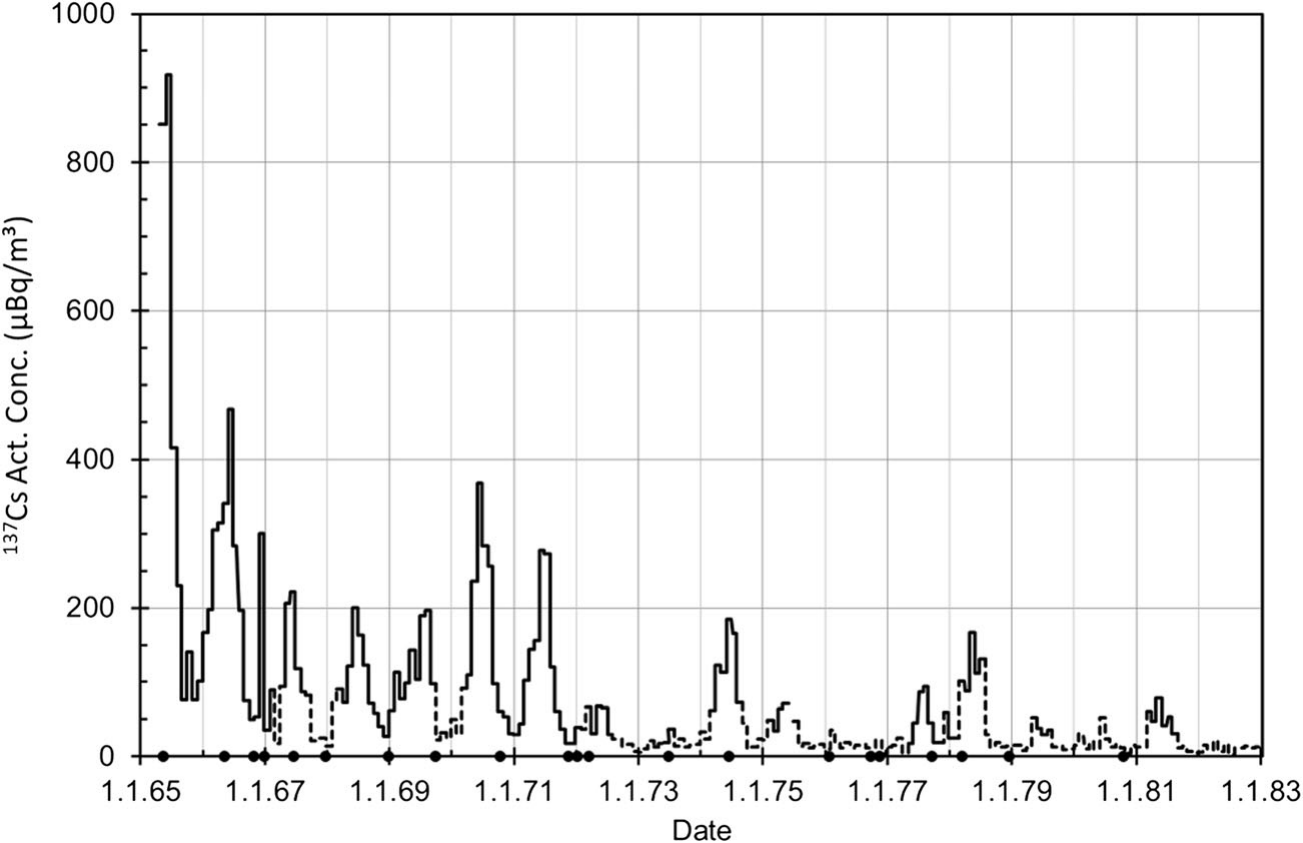
Monthly mean values of ^137^Cs activity concentration (μBq/m^3^) in Rovaniemi in 1965–1982. The dotted parts of the curve represent “half of MDA” concentrations. The dots on the *x*-axis represent atmospheric nuclear tests performed by People’s Republic of China

Monthly ^137^Cs activity concentrations fell below the detection limit quite rapidly, already in 1967, especially during autumn and winter months (Fig. 11). An interesting detail is that there seems to be no temporal correlation between the observed concentrations and the atmospheric nuclear tests performed by People’s Republic of China. Evidently, the resulting stratospheric injection of ^137^Cs varied case by case depending on season, burst height, and energy released. Globally, the fission product contamination in the ground-level air from the stratosphere was rather evenly distributed. Therefore, it is understandable that the values of ^137^Cs activity concentrations are very similar in Rovaniemi and in Helsinki. For example, in January–June 1967, the ^137^Cs activity concentration in Helsinki was 93 μBq/m^3^ (± 15%) and 56 μBq/m^3^ (± 11%) in July–December (Jaakkola et al. 1979). The results of Rovaniemi also repeat the slightly higher values of January–June 1970 (150 μBq/m^3^ ± 15%) and July–December 1970 (100 μBq/m^3^ ± 15%) in Helsinki.

### Stratosphere as a source of fission products in the ground-level air

After the first atmospheric thermonuclear detonations in the 1950s, it was discovered that the explosion plumes rose through the tropopause into the stratosphere. In the troposphere, the residence time of radionuclides released in the explosions is only a few weeks. However, in the stratosphere, the residence time of the radioactive debris varies from several months to a couple of years. During this time, the debris is quite evenly distributed all over the stratosphere. The transfer of stratospheric air masses containing radionuclides into the troposphere transports artificial radionuclides gradually into the ground-level air. Numerous reports have been published about the phenomenon that occurs especially in spring (e.g., Staley 1962; Warmbt 1966; Feely et al. 1966; Danielsen 1968). Airborne fission products from the nuclear tests have been used as tracers in meteorological research (Machta 2002).

**Fig. 12:**
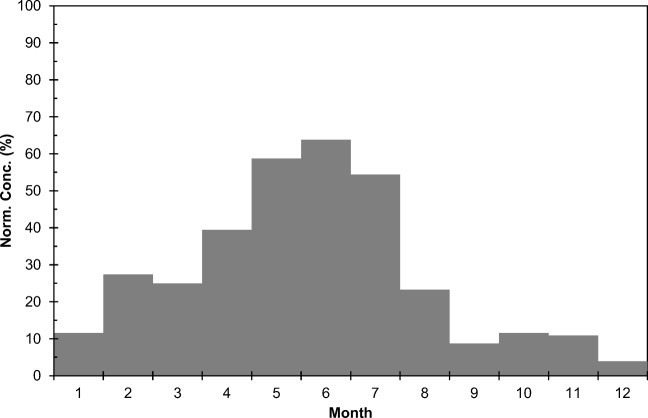
Seasonal variation of normalized fission product activity concentrations in Rovaniemi in 1965–1983

**Fig. 13:**
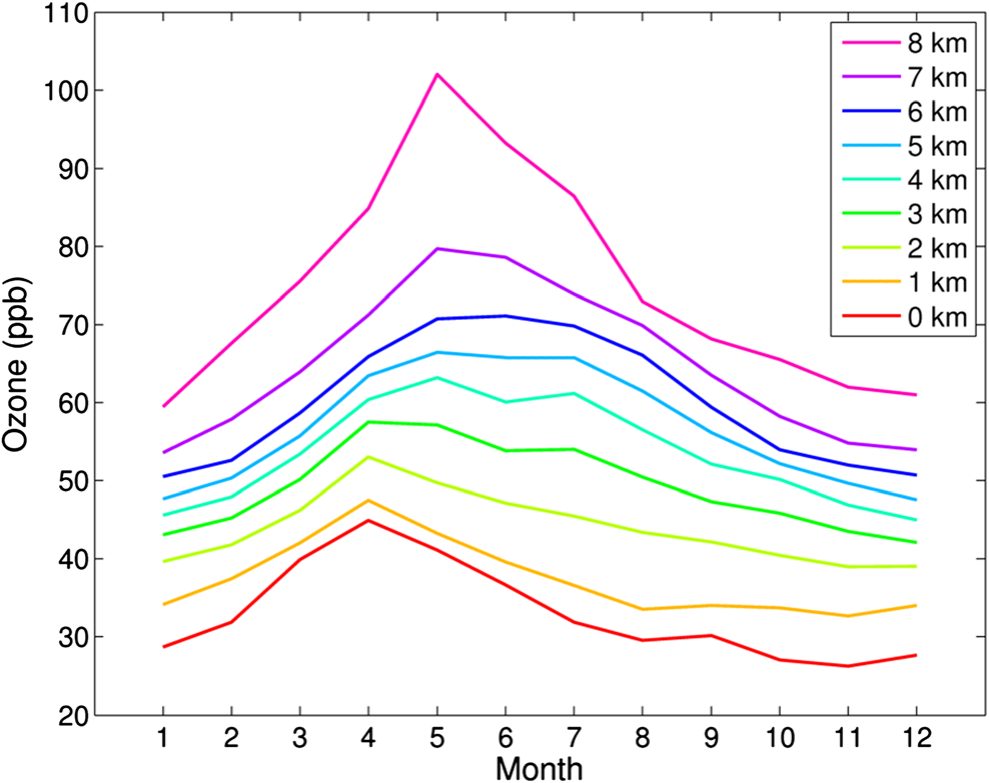
Seasonal variation of tropospheric ozone concentration at Sodankylä, Finland. The data is based on ozone soundings (Kivi et al. 2007; Christiansen et al. 2017)

The seasonal variation of fission product concentration between 1965 and 1981 was calculated by first subtracting monthly ^210^Pb activity concentrations at Sodankylä from the monthly average total beta activity concentrations. The ^210^Pb results from Sodankylä are representative also for Rovaniemi because the ^210^Pb content of the air varies little in the scale of a few hundred kilometers (Mattsson et al. 1996). To cancel the effect of year to year variation of fission product concentration, normalized fission product concentrations were calculated. The highest monthly concentration of each year received a value of 100% and the other months correspondingly a smaller value. Finally, the average values for each month were calculated. The highest normalized concentration values are found in May–July and the lowest ones in autumn and early winter (Fig. 12). The seasonal variation of fission product concentration is quite similar to the cosmogenic radionuclide ^7^Be. In Southern Finland, the highest ^7^Be activity concentrations in ground-level air occur in May and June (Aaltonen et al. 2001). However, one has to bear in mind that a significant fraction (25–33%) of ^7^Be is produced in the troposphere and not transported from the stratosphere (Johnson and Viezee 1981; Leppänen et al. 2012). The seasonal variation of tropospheric ozone concentration measured with balloon soundings at Sodankylä resembles the behavior of fission product concentration too (Fig. 13). In case of ozone, its concentration in the troposphere is affected by three factors: transport from the ozone layer in the stratosphere, losses in chemical reactions in the troposphere, and photochemical production from atmospheric hydrocarbons and nitrogen oxides closer to the ground.

**Fig. 14:**
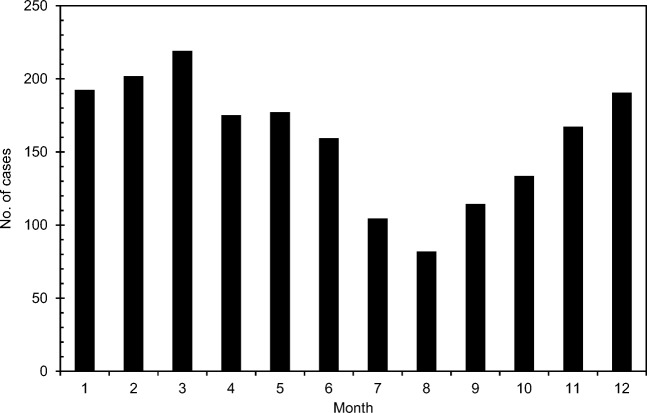
Seasonal variation of cases with stratospheric air on the 290 hPa pressure level

To further study the effect of stratospheric air masses moving into the troposphere, a set of potential vorticity (PV) values was calculated by using wind, temperature, and surface pressure fields from European Centre for Medium-Range Weather Forecasts (ECMWF) for the years 2000–2015. The unit of potential vorticity is m^2^ s^−1^ K kg^−1^. According to definition, 1.0 × 10^−6^ m^2^ s^−1^ K kg^−1^ is one potential vorticity unit (1 PVU). Potential vorticity increases when the stability of the atmosphere increases. It also increases from the troposphere to the stratosphere (e.g., Holton et al. 1995). The location of tropopause is usually defined by using a PV value of 2 PVU. The Brewer-Dobson circulation includes rising motion in the tropics and descending motion in the extratropics. This downward transport in the extratropics through the tropopause transports air and atmospheric constituents across the tropopause and is an important source of tropospheric ozone and other compounds, e.g., ^7^Be (Paatero and Hatakka 2000; Baray et al. 2017). Potential vorticity values at the pressure level of 290 hPa were computed in 1.5° intervals between the latitudes 45°N and 70.5°N along the meridian 20°E. Next, we calculated the number of days when the PV was more than 3 PVU at the 290 hPa pressure level, i.e., how often there was stratospheric air at that pressure level under the tropopause. Figure 14 reveals that most often this occurs in March. The occurrence rate decreases later in spring and drops to its seasonal minimum in late summer. These results are in agreement with the seasonal variation of fission product concentration as it takes a couple of months for the stratospheric species in the upper troposphere to reach ground-level air (Liang et al. 2009).

## Conclusions

Long time series collected at the same sampling site since 1965 has provided a continuous view to atmospheric radioactivity in Finnish Lapland through the decades covering various nuclear events. In this study, contamination from different sources in surface air of Rovaniemi was found based on concentrations of ^137^Cs, ^90^Sr, and total beta activity in the air filter samples. These contamination sources include global fallout from nuclear weapon testing, Chernobyl, and Fukushima accidents, leakages from underground nuclear tests performed in Semipalatinsk in 1966 and in Novaya Zemlya in 1987, and atmospheric nuclear tests performed in 1965–1980. Lighter radionuclides like ^137^Cs have been transported from the destroyed Chernobyl reactor to Finnish Lapland whereas intermediate radionuclides like ^90^Sr have not reached this region.

After the Fukushima accident, there have been some radionuclide release events, e.g., the emission of ^131^I from the Institute of Isotopes Ltd., Hungary, in September–November 2011. This release was not detectable in the total beta activity measurements in Rovaniemi due to the relatively high natural background radioactivity level formed mainly by ^210^Pb compared with the trace levels of ^131^I in the surface air (Tichý et al. 2017). The analyses of ^137^Cs and ^90^Sr content of aerosol samples ended in 2011, although the sampling program of Finnish Meteorological Institute still continues in Rovaniemi. Therefore, later radionuclide releases like ^131^I detected widely in Europe in January–February 2017 as well as ^106^Ru observed in Russia and Europe in September–October 2017 remain unfortunately beyond the scope of this study.

Deeper insight to stratosphere-troposphere exchange of radionuclides and seasonal changes in radionuclide concentrations in the surface air was obtained by using ozone observations and meteorological models, which provided a deeper insight into the often superficially explained phenomenon.
